# Speckle tracking echocardiography in mature Irish Wolfhound dogs: technical feasibility, measurement error and reference intervals

**DOI:** 10.1186/1751-0147-55-41

**Published:** 2013-05-16

**Authors:** Ulrik Westrup, Fintan J McEvoy

**Affiliations:** 1 Department of Small Animal Clinical Sciences, Faculty of Life Sciences, University of Copenhagen, Dyrlaegevej 16, Frederiksberg C, DK-1870, Denmark

**Keywords:** Canine, Speckle tracking echocardiography, Strain, Left ventricle, Torsion

## Abstract

**Background:**

Two-dimensional strain measurements obtained by speckle tracking echocardiography (STE) have been reported in both humans and dogs. Incorporation of this technique into canine clinical practice requires the availability of measurements from clinically normal dogs, ideally of the same breed, taken under normal clinical conditions.

The aims of this prospective study were to assess if it is possible to obtain STE data during a routine echocardiographic examination in Irish Wolfhound dogs and that these data will provide reference values and an estimation of measurement error.

**Methods:**

Fifty- four healthy mature Irish Wolfhounds were used. These were scanned under normal clinical conditions to obtain in one session both standard echocardiographic parameters and STE data. Measurement error was determined separately in 5 healthy mature Irish Wolfhounds.

**Results:**

Eight dogs were rejected by the software algorithm for reasons of image quality, resulting in a total of 46 dogs (85.2%) being included in the statistical analysis. In 46 dogs it was possible to obtain STE data from three scanning planes, as well as to measure the rotation of the left ventricle at two levels and thus calculate the torsion of the heart. The mean peak radial strain at the cardiac apex (RS-apex) was 45.1 ± 10.4% (n = 44), and the mean peak radial strain at the base (RS-base) was 36.9 ± 14.7% (n = 46). The mean peak circumferential strain at the apex (CS-apex) was -24.8 ± 6.2% (n = 44), and the mean peak circumferential strain at the heart base (CS-base) was -15.9 ± 3.2% (n = 44). The mean peak longitudinal strain (LS) was -16.2 ± 3.0% (n = 46). The calculated mean peak torsion of the heart was 11.6 ± 5.1 degrees (n = 45).

The measurement error was 24.8%, 26.4%, 11.5%, 6.7%, 9.0% and 10 degrees, for RS-apex, RS-base, CS-apex, CS-base, LS and torsion, respectively.

**Conclusions:**

It is concluded that this technique can be included in a normal echocardiographic examination in large breed dogs under clinical conditions. The usefulness of the reference values reported here, given their wide normal range, will ultimately be determined by the values that are obtained from a large numbers of diseased dogs.

## Background

The Gold standard for describing myocardial movement in three dimensions has, until recently, been MRI tagging in humans and sonomicrometry in dogs [1]. Both methods have shown promise in the study of LV motion and in calculating LV torsion, but they are limited in availability. Both techniques require anaesthesia and they are time-consuming and expensive [1].

Currently, other methods are available. These methods have been validated against MRI tagging and sonomicrometry and have been found to be acceptable [1]. One such method is speckle tracking echocardiography (STE) [1-3]. The principles behind the speckle tracking technique are based on the ability of software algorithms to recognize a pattern of speckles in a marked area on an image and then follow this area’s movement during a cardiac cycle. Speckles arise from interaction between the ultrasound beam and sub-resolution scatterers. Changes in speckles are strongly correlated with tissue movement, and if retained in the signal, they can be used to track motion.

STE can measure “regional strains”, which are dimensionless measurements of deformation that are expressed as a fractional or percentage change from an object’s original dimension:

$$\mathrm{Strain}\left(S\right)=\frac{l-l_{0}}{l_{0}}=\frac{\mathrm{\Delta l}}{l_{0}},$$

where *l* is the instantaneous length, *l*_*0*_ is the initial length, and Δ*l* is the change in length.

Three scanning planes are identified for strain measurements. They result in radial, circumferential and longitudinal measurements referring to changes in ventricle wall thickness, ventricular circumference and the apex-to-base dimension, respectively.

**Figure 1:**
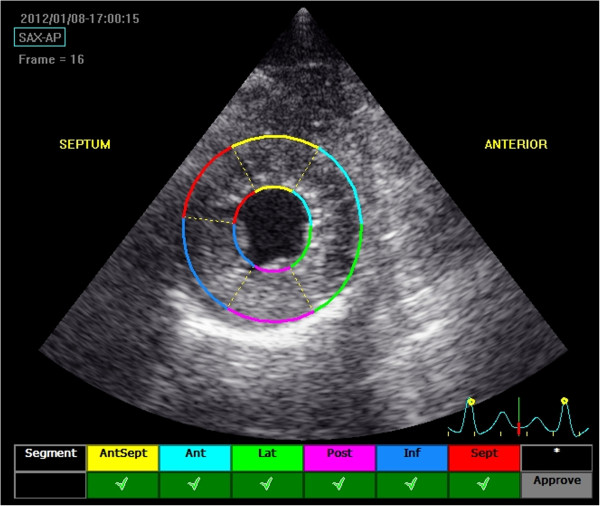
**Speckle tracking echocardiography at the level of the apex.** The software algorithm automatically separates the LV short-axis into 6 myocardial segments to include the interventricular septum and the LV free wall. The tracking approval of each individual myocardial segment is displayed on the screen.

**Figure 2:**
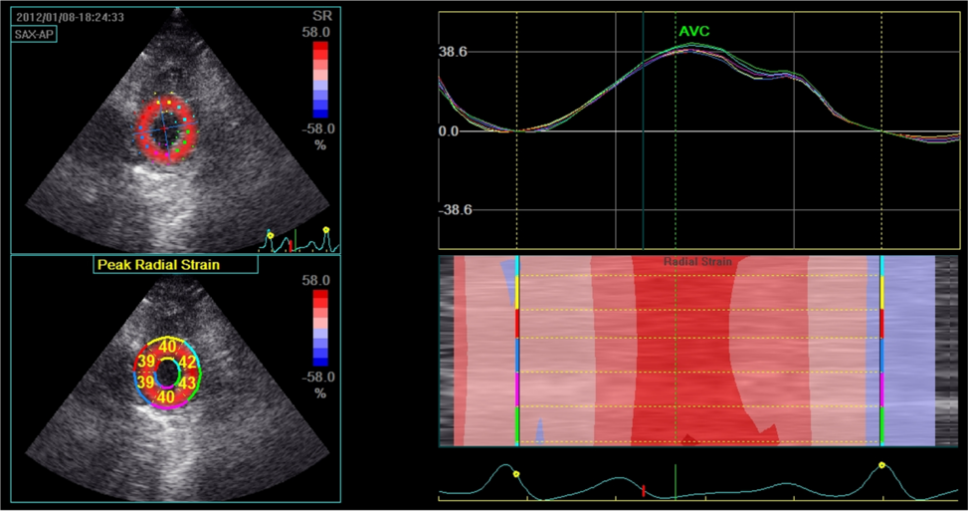
**Radial strain at the level of the apex.** Top left: Peak systolic apical radial strain displayed using a colour map. Bottom left: Peak systolic apical radial strain displayed as a percentage for each individual segment. Top right: Left ventricular radial strain versus time curves corresponding to the 6 myocardial segments, Y-axis unit is %. Right bottom: Two-dimensional and M-curve colour-coded views show positive strain during systole.

**Figure 3:**
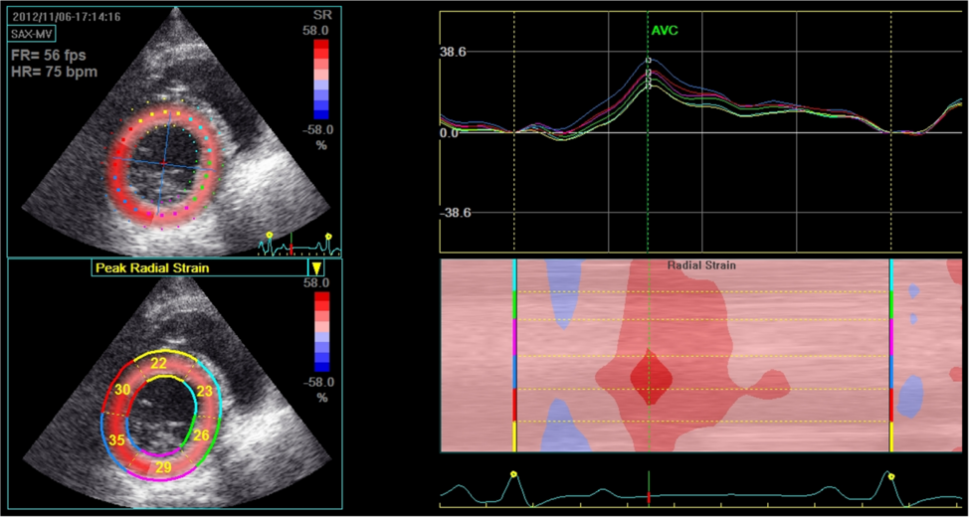
**Radial strain at the level of mitral valve.** Top left: Peak systolic apical radial strain displayed using a colour map. Bottom left: Peak systolic apical radial strain displayed as a percentage for each individual segment. Top right: Left ventricular radial strain versus time curves corresponding to the 6 myocardial segments, Y-axis unit is %. Right bottom: Two-dimensional and M-curve colour-coded views show positive strain during systole.

**Figure 4:**
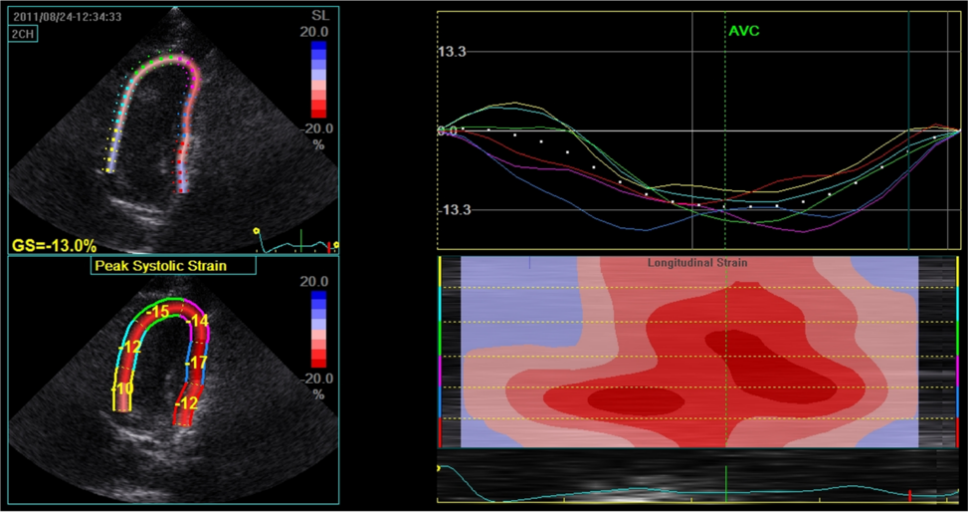
**Speckle tracking echocardiography of the left parasternal apical 4**-**chamber view of the left ventricle.** Top left: Colour-coded strain of the left ventricle and the average peak systolic strain map (percentage), GS = Gobal Strain. Bottom left: Peak systolic strain of each segment. Top right: Left ventricular longitudinal strain versus time curves corresponding to the 6 myocardial segments. Right bottom: Two-dimensional and M-curve colour-coded views showing negative strain during systole.

**Figure 5:**
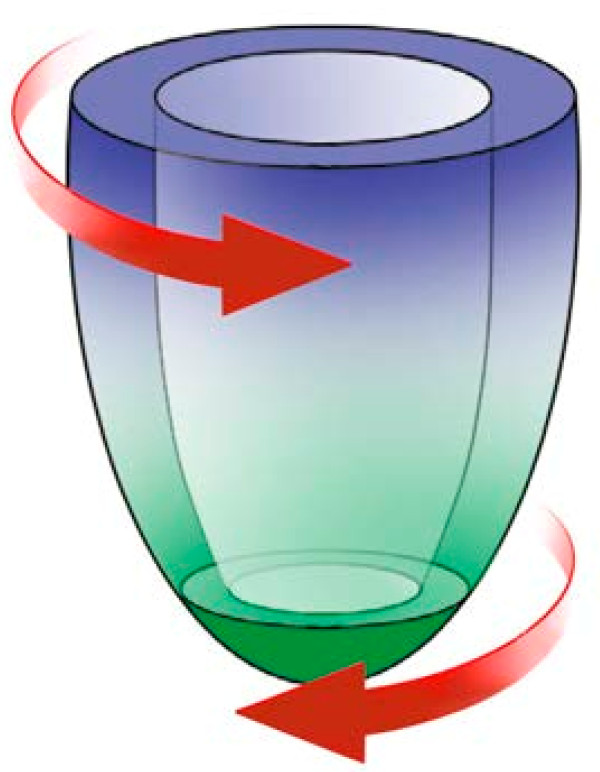
**Schematic representation of the left ventricle.** The levels where the measurements were obtained and the direction of rotation. The basal rotation is subtracted from the apex rotation, providing the torsion of the LV.

**Figure 6:**
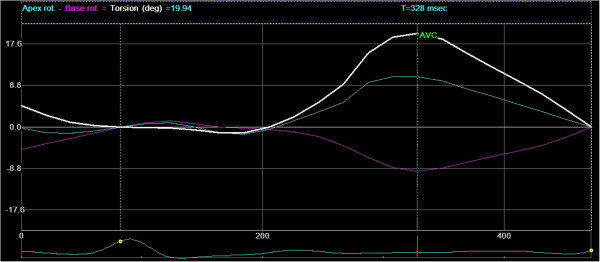
**Rotation versus time curves.** Rotation versus time curves from the apex and base levels and the time curve of basal rotation subtracted from the apex rotation, which provides the twist of the heart in degrees. AVC = Aortic Valve Closure, X-axis unit is millisec. And Y-axis unit is %.

During systole, radial strain (RS) is expected to be a positive value, (Figures 1, 2 and 3). Circumferential strain (CS) is expected to be negative and longitudinal strain (LS) is normally negative due to a decrease in length of the left ventricle in systole shown in human studies [4] (Figure 4). Calculation of the extent of LV torsion requires short axis speckle tracking data at the level of the apex and at the level of the mitral valves (base) (Figure 5). Software can calculate the rotation at both levels. Viewed from apex to base, counter-clockwise rotation, in degrees, is expressed as a positive value and clockwise rotation, in degrees, as a negative value. Peak LV torsion is then defined as peak systolic basal rotation minus peak systolic apical rotation (Figure 6).

STE mapping of cardiac movement over a period has been validated in humans for the assessment of various heart diseases, including ischaemic lesions and dilated cardiomyopathy (DCM) [5,6]. The technique has been used to detect myocardial ischaemic memory, in a canine model [7]. It is anticipated that STE could be of value in canine cardiology. There is a need for such novel parameters in this area since the standard echocardiographic data-set in current use is poor both for the early detection of DCM and for use in prognosis.

The normal reference ranges for a breed that has a high prevalence DCM, a cardiac disease relevant to the technique, obtained under normal clinical imaging conditions will help determine if STE measurements can be used to meet these diagnostic and prognostic gaps.

The aims of this study were to obtain LV systolic strain and torsion data using STE in a clinical setting, from healthy, mature Irish Wolfhound dogs, to report reference intervals and to estimate the measurement error.

## Methods

### STE imaging and calculation of reference values

All of the dogs presented for inclusion (n = 54) were participants in a dilated cardiomyopathy (DCM) screening program in Denmark and Norway, where breeders are recommended to submit their dogs for an annual ECG and echocardiographic examination. The dogs were accustomed to echocardiographic examination. Dogs younger than one year old were excluded. All were healthy and free of medication and had no history of heart or respiratory disease. Informed owner’s consent for each dog was obtained before inclusion. A clinical examination, ECG, in right lateral recumbency according to normal procedures [8], and a standard echocardiographic examination for DCM (with ECG tagging) were performed. The echocardiographic examination included, as a minimum, the right parasternal short-axis (transverse) view of the heart base (at the level of the cordae tendinae and at the aortic valve cusps) and the right parasternal long axis. These views were used as appropriate [9], to evaluate the left atrium-to-aorta ratio, LV M-mode measurements of wall and chamber sizes, the percentage of fractional shortening and the E-point to septal separation. Dogs showing signs of pathological arrhythmia on ECG or during echocardiographic examination were excluded from the study. The study required that all of these sonographic parameters fall within accepted ranges [9,10].

STE echocardiographic images were obtained with the dogs positioned primarily in right lateral recumbency, using right parasternal short-axis views at the level of the apex and at the base of the LV, and left parasternal apical four-chamber views. The left parasternal apical four-chamber view was obtained with the dogs positioned in left lateral recumbency if the image from the right lateral recumbent view was considered inadequate. All of the dogs were imaged without sedation. One complete image set per dog, consisting of at least three consecutive cardiac cycles, was stored for later analysis.

All of the imaging exams were performed by the same experienced sonographer (UW), using either a General Electric Vivid 7 BT 08, M4S transducer^a^ or a Vivid E9 BT 11 M5S transducer.^a^ The post-processing calculation of the LV strain and torsion was performed using software available as an option in EchoPac 110.1.2.^b^ “Manual marking” of the myocardium was performed as described in previous studies [11,12]. The post-processing software divides the myocardium into six segments, and the calculated strain values represent an average of the six segments measured. The means of these calculated strain values for all of the dogs included are reported here. Given that the measurements were obtained from three views — right parasternal short-axis view at the level of the apex and at the base of LV (mitral valve level) and a left parasternal apical four-chamber view — a total of 18 segments of the myocardium were tracked. A minimum of 47 frames per second was used for all acquisitions. Dogs in which one or more segments could not be tracked by the software were eliminated from the study.

The following parameters were extracted from the images: radial and circumferential strain at the level of the cardiac apex, radial and circumferential strain at the level of the cardiac base (mitral valve level), longitudinal strain and the rotation of the heart at both the apex and base to calculate cardiac torsion.

### Measurement error

The measurement error of the STE variables is reported as the repeatability (RP) statistic for each measurement [13]. Five adult, healthy Irish wolfhounds were used to determine this error. Each dog underwent an echocardiographic examination, following the same protocol for strain and torsion measurements as described above. Echocardiographic examinations were repeated five times on each dog, with the dog positioned on the examination table for approximately one minute between each repeat scan.

### Statistical analysis

#### Reference values

All of the plots and statistical tests were performed using R statistical software [14]. ^c^ Reference intervals were determined according to the protocol published by the Association of Veterinary Clinical Pathology Reference Standard Committee [15]. Histograms and box and whisker plots were created for each parameter and were viewed for evidence of normal distribution and for the presence of possible outliers. For each parameter, a formal test for outliers was performed using the “scores” function in R, with a probability of p = 0.99 used as a threshold. If an outlier was identified, it was removed, and the revised data set was reassessed for outliers. This process was repeated until no outliers were detected.

The data with outliers removed were then checked for normal distribution using the Shapiro-Wilk test. Possible bias in the data, with regard to age and heart rate, was assessed using a linear regression model, and with regard to gender using Student’s t-test, the null hypothesis was rejected in each test for p < 0.05.

Means and standard deviations (SD) were calculated once the data were cleared of outliers and were confirmed to have a normal distribution and to be free of age, heart rate and gender bias. The upper and lower reference intervals are determined as the mean plus 2SD and mean minus 2SD for each parameter. The 95% confidence interval (CI) for these reference ranges was calculated. This indicates the degree of uncertainty in the range values and is a function of the sample size. It is calculated as the standard deviation of the observations times the square root of the ratio, 3/n, where n is the number of observations.

#### Measurement error

Measurement error, described by within-subject standard deviation, was determined according to established protocols [15]. Briefly, analysis of variance was used to estimate the mean square residual of the replicate data in five dogs. The square root of this value is the within subject standard deviation (sw). Repeatability, a simple product of the within subject standard deviation (2.77 times sw), is an indicator of measurement error. It has dimensions that are the same as those of the measurements it describes. Repeatability, calculated in this manner, provides the expected maximum difference between two measurements of the same subject in 95% of pairs of observations.

The analysis assumes that the standard deviation of the repeat measurements is independent of the magnitude of the measurement. For all parameters, this assumption was checked graphically and using Kendall’s tau rank coefficient. A separate indicator of variability, the coefficient of variation CV% (standard deviation divided by the mean) is also reported.

## Results

### Reference values

A total of 54 dogs were presented for inclusion. Eight dogs were rejected by the software algorithm for reasons of image quality, resulting in a total of 46 dogs being included in the study. Fourteen were male, and 32 were female. The mean age was 3.95 ± 1.74 years [range: 1.00-8.25], and the mean weight was 66.09 ± 9.17 kg [range: 53-85], both parameters were normally distributed.

The total extra time used per dog to obtain the 3 views for STE was approximately 2 minutes. The post-processing calculation time was approximately 5 min. per dog.

**Figure 7:**
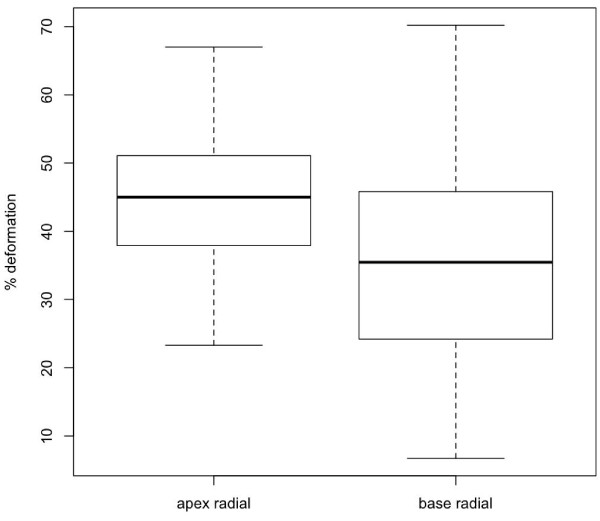
**Box**-**and**-**whisker plots of peak systolic radial strain at apex and base of left ventricle.** Data showing 2.5, 25, 50 (median), 75 and 97.5 cumulative centiles.

**Figure 8:**
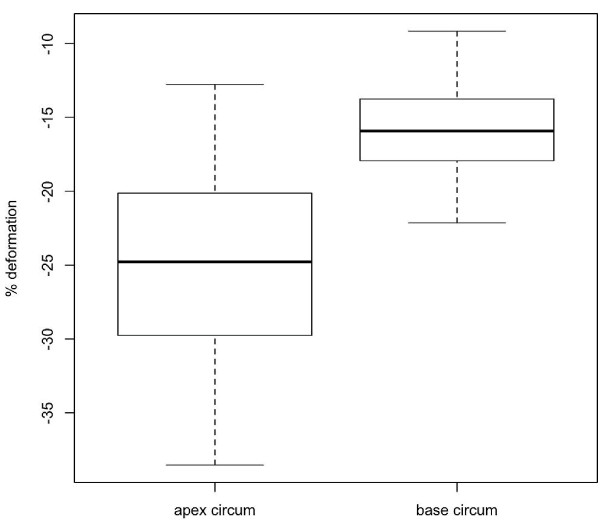
**Box**-**and**-**whisker plots of peak systolic circumferential strain at apex and base of left ventricle.** Data showing 2.5, 25, 50 (median), 75 and 97.5 cumulative centiles.

**Figure 9:**
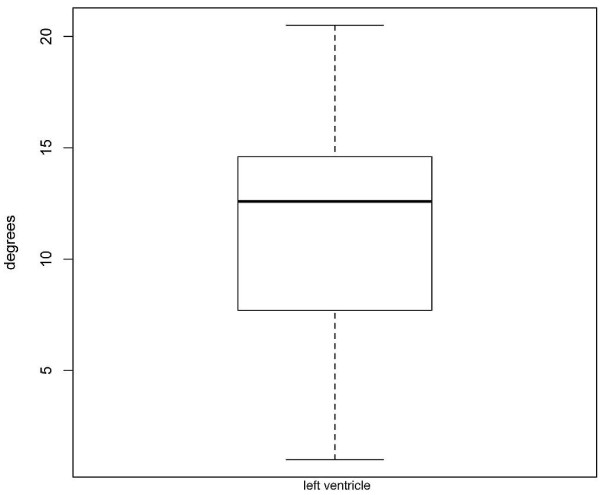
**Box**-**and**-**whisker plot of peak systolic longitudinal strain of left ventricle.** Data showing 2.5, 25, 50 (median), 75 and 97.5 cumulative centiles.

**Figure 10:**
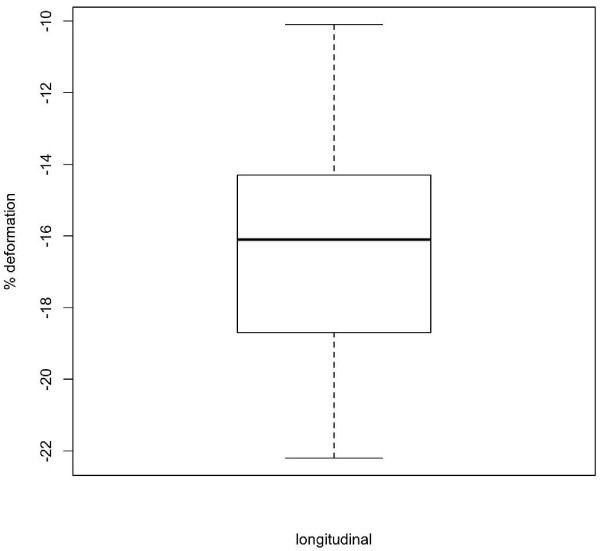
**Box**-**and**-**whisker plot of peak systolic torsion of left ventricle.** Data showing 2.5, 25, 50 (median), 75 and 97.5 cumulative centiles.

There was a total of six outliers, two for the radial measurements, two for the circumference measurements at the apex level, and one each for the circumferential strain measurements at the base and for the twist measurements. The outliers were removed before the reference intervals were calculated. A box and whisker plot for each parameter (with outliers removed) is shown in Figures 7, 8, 9 and 10. For all of the parameters, there were no significant relationships regarding age, gender or heart rate when tested in the regression model or using Student’s t-test as appropriate.

The null hypothesis in the Shapiro-Wilk test, i.e., that the samples come from a normal distribution, was not rejected for any of the parameters. The 95% reference interval was calculated as the mean ± 2SD. This interval, the mean of the data and the number of samples for each parameter are provided in Table 1.

**Table 1:** **Left Ventricle peak systolic strain and twist measurements obtained by speckle tracking echocardiography** (**STE**), **measurement error and coefficient of variation**

	**Mean (SD)**	**[Reference interval]**	**N**^**a**^	**CI**^**b **^**95%**	**M. Error**^**c**^	**CV%**^**d**^
Peak apical systolic radial strain (%)	45.1 (10.4)	[24.3 to 65.9]	44	10.8	24.8	7.1
Peak apical circumferential strain (%)	24.8 (6.2)	[-12.8 to -38.5]	44	6.5	11.5	3.0
Peak basal systolic radial strain (%)	36.9 (14.7)	[7.5 to 66.3]	46	15.0	26.4	7.1
Peak basal circumferential strain (%)	15.9 (3.2)	[-22.3 to -9.5]	44	3.3	6.7	6.8
Peak longitudinal strain (%)	16.2 (3.0)	[-22.2 to -10.2]	46	3.0	9,0	5.4
Left ventricle torsion (degrees)	11.5 (5.1)	[1.3 to 21.7]	45	5.2	10.0	3.4

^a^ N, numbers of dogs.

^b^ CI, confidence interval (calculated as standard deviation of the observations times the square root of the ratio, 3 dividend by the number of observations).

^c^ M. Error, refers to the repeatability statistic. Measurement error has dimensions that are the same as those of the measurement it describes.

^d^ CV% Coefficient of variation. For clarity the absolute values for CV are given here.

### Measurement error

Measurement errors, expressed as coefficient of variation and the repeatability statistic, are provided in Table 1. In general, the reference ranges for the STE measurements reported above were wide. The repeatability data accounted for some, but not all, of this range.

## Discussion

The determination of reference values is a complex procedure. Veterinary studies are often limited by small population sizes, due especially, in clinical settings, to economic and availability considerations. The American Society of Veterinary Clinical Pathology (ASVCP) addressed these issues in recently published guidelines for the determination of reference intervals in veterinary species [15]. We used these guidelines in an attempt to achieve good practice and thus provide reliable clinical data.

The criterion that all 18 segments in our STE exam yield strain measurements was also adopted in this study to provide reliable data. This strict inclusion criterion resulted in the exclusion of eight out of 54 dogs (14.8%). In a previous study using STE to measure left ventricle radial strain in dogs, 5% of the segments showed poor tracking [11]. In human measurements, the probability of tracking a healthy segment has been shown to lie at approximately 97% but to drop to 80% in diseased segments [16].

The values obtained in this study relate to one breed. Mean peak LV systolic radial strain values have been published in 37 healthy dogs from 20 different breeds [12]. The LV measurements in that study were only taken at the papillary muscle level. Our results of STE measurements taken at the heart base and at the apex are therefore not directly comparable to the measurements taken at the papillary muscle level in that study but they are necessary for us to be able to measure the torsion of LV.

Longitudinal strain in our study was -15.9 ± SD 3.2%. Only the left parasternal apical four-chamber view, with a total of six segments, was used in our study. According to the guidelines of the American Society of Echocardiography [17], three apical views are desirable for the measurement of longitudinal strain. This requirement subdivides the LV into 18 segments. The global average longitudinal strain, based on these 18 segments in humans, was reported to be −20.6 ± SD 2.6% [18]. That study, however, showed a significant difference among measurements obtained from the three left parasternal apical views. In a veterinary setting, however, obtaining three different strictly defined apical views is problematic simply because of the physical conditions working with awake healthy animals. It remains to be seen whether the lower longitudinal strain values reported here were related to the use of a single apical view, constitute a general trend in dogs, indicate a distinct feature of the Irish Wolfhound or are a combination of the three.

Torsion in our population was reported as 11.6 ± SD 5.1 degrees. This result is higher than that reported (8.4 ± 3.8 degrees) in a study of 35 healthy dogs of different breeds [11]. Our population differed quite substantially from the population in that study, which included dogs aged less than one year old that had a mean body weight of 18.4 kg (compared to 66.1 kg in this study). Dogs younger than one year of age were excluded from this study because it is known that, in humans, apical rotation of the LV changes markedly during the first year of life, and the twist can be close to zero in very young individuals [19]. If the same effect were seen in young dogs, their inclusion in this study would have added bias to the data and would have resulted in lower mean torsion measurements. It is possible that a different set of reference intervals would be required for immature dogs. In the adult human population, twist measurements have been correlated with age [20,21], a finding we were not able to reproduce in our study population. Notwithstanding the effect of age mentioned above, the average twist in the adult human heart was reported to be 10.5 ± SD 2.7 degrees [21], which is similar to our value of 11.6 ± SD 5.1 degrees. An age-related effect was seen in the study of 35 dogs mentioned above [11]. That study showed a positive correlation between age and peak end systolic rotation at the base [11]. Again, that study and the current study differ in that the site of measurement at the heart base and the study population are not comparable.

The magnitudes of the ranges for all STE strain measurements and for torsion were larger compared to those of a previously published study [11]. The repeatability statistic reported here indicates that within subject standard deviation or repeatability can account for some, but not all, of the ranges of values encountered. For example, the reference range for peak systolic radial strain at the apex (23.3-67.0%) spanned some 43.7 percentage points. The repeatability statistic for this parameter was 24.8 percentage points. It indicates the maximum difference expected between two measurements on the same animal due to measurement error. For this parameter, because the repeatability was less than the reference range, it follows that variation between individuals (i.e., between subject difference) contributed to the range reported here. It is highly unlikely that two measurements from the same animal would span the full reference range. It follows that some of the range encountered in this study was the result of between subject differences.

The usefulness of the reference values reported here, given their wide normal range, will ultimately be determined by the values that are obtained from diseased dogs. A wide range of normal values is not of any concern if a large proportion of abnormal dogs have values that fall outside this range. We are aware that our population could include dogs with occult DCM not detectable clinically or on routine echocardiographic examination. Occult DCM is of course a concern of great importance when our aim is to provide reference values for a healthy population of mature Irish Wolfhounds. It has been shown in a Great Dane with no clinical findings but equivocal findings of DCM obtained by conventional 2-dimensional and M-mode echocardiography where few ventricular premature complexes also where noted during the examination [22] that Tissue Doppler Imaging (TDI) revealed abnormal myocardial velocity gradients, related to decreased endocardial velocities [22]. There are however, no standard normal TDI values for Irish Wolfhound dogs, so it was not possible for us to use TDI to exclude occult DCM dogs from our population. Inclusion in our study required normal conventional 2- dimensional and M- mode echocardiography. None of the dogs had equivocal parameters and none showed pathological arrhythmias. This does not mean that occult DCM, beyond the reach of current diagnostic aids, did not exist in our population of dogs. Longitudinal studies would go some way to address this issue by seeking trends in STE data that are characteristic of dogs that go on to develop clinical disease.

Even though the STE has been shown to be both repeatable and reproducible for assessing systolic LV torsional deformation having within day and between day variability’s less than 10% for radial strain measurements [11,12], inter- and intra observer data, which was not available for this study, would add important knowledge on the technique's usefulness.

In humans, the availability of reference values for STE in healthy populations has made it possible to quantify the severity of heart disease. It has been shown that apical rotation is a quantitative marker in patients with dilated cardiomyopathy. Using the STE technique, targets can be set for preventive strategies that might reduce the prevalence of heart failure in this group of patients [6,23,24].

STE is still a novel, but very promising, technique. The measurements are not very time-consuming to obtain, but they are nonetheless demanding to acquire in that high-quality images, high-end equipment and dedicated proprietary software, such as EchoPac, are required.

Despite these considerations, it is likely that the technique will become more widely available and will be the subject of further investigation in veterinary echocardiology. STE has shown promise in several pathological human conditions, and it remains to be seen whether this promise will also be seen in veterinary medicine.

Some limitations of this study can be identified. The filtering algorithms used by software in tracking the speckles from the myocardium can vary from vendor to vendor. It is thus unclear how the values from different scanners and software versions would compare. Cross-platform comparisons and a clear definition of global and regional normal values are essential for broader application of STE [25].

The measurement of LV longitudinal strain was obtained from the left parasternal apical four-chamber view with the dog positioned in right lateral recumbency. Obtaining such images (in dogs with a mean body weight of 66 kg) was demanding on both the equipment and the sonographer. In approximately one out of 5 cases, the dog was moved from right to left lateral recumbency to improve image quality for this view. This manoeuvre could have introduced a source of measurement error. However, it should be noted that the reference range is not unusually wide for this parameter.

## Conclusions

From this study, it can be concluded that it is possible to obtain STE measurements, including radial, circumferential, and longitudinal strain and the twist of the left ventricle, in large-breed dogs under clinical conditions. The normal reference values provided here for Irish Wolfhound dogs, a breed with a genetic disposition for cardiac disease, could be useful in guiding the interpretation of STE measurements in clinical practice.

## Endnotes

^a^GE Healthcare, Copenhagen, Denmark.

^b^EchoPac PC 110.1.2 software for Vivid 7 and Vivid E9 GE Healthcare, Copenhagen, Denmark.

^c^R version 2.14.1 (2011-12-22) 2011 The R Foundation for Statistical Computing ISBN 3-900051-07-0.

## Abbreviations

AO: Aorta; BSA: Body surface area; CS: Circumferential strain; DCM: Dilated cardiomyopathy; ECG: Electro cardiography; EPSS: E-point to septal separation; FS: Fractional shortening; LA: Left atrium; LS: Longitudinal strain; LV: Left ventricle; RS: Radial strain; SD: Standard deviation; STE: Speckle tracking echocardiography; TDI: Tissue doppler imaging.

## Competing interests

Both authors declare that they have no competing interests.

## Authors’ contributions

UW headed the study, performed the echocardiographic examination and the data processing on the Echopack and was main responsible for drafting the manuscript. FJM contributed with substantial statistic work as well as participating in the design and writing the manuscript. Both authors read and approved the final manuscript.
